# Navigating the nutritional paradox: The impact of sustainable development targets on childhood wasting and overweight prevalence

**DOI:** 10.1371/journal.pgph.0003335

**Published:** 2024-06-21

**Authors:** Mukhtar A. Ijaiya, Seun Anjorin, Olalekan A. Uthman

**Affiliations:** 1 Data-Lead Africa, Central Business District, Abuja, Federal Capital Territory, Nigeria; 2 Big Data Institute, Nuffield Department of Population Health, University of Oxford, Oxford, United Kingdom; 3 Division of Health Sciences, Warwick Centre for Global Health, Warwick Medical School, University of Warwick, Coventry, United Kingdom; Wageningen University & Research, NETHERLANDS

## Abstract

In 2015, the United Nations member states endorsed the 2030 Agenda for Sustainable Development to chart a path towards a better future for all. Childhood malnutrition, particularly wasting, remains a critical global health challenge, disproportionately affecting children under five in low- and middle-income countries. This study evaluates the impact of achieving selected Sustainable Development Goal (SDG) targets on reducing childhood malnutrition, with a specific focus on wasting and overweight. Utilizing multi-country DHS datasets, this study analyzed data from 138,782 children under five across 27 countries, nested within 13,788 neighborhoods. We simulated the predicted prevalence of wasting and overweight as selected SDG-related health inputs and determinant indicators reached their target values. Our findings reveal a baseline prevalence of 6.3% for wasting and 4.3% for overweight among the children studied. Progress towards the SDGs can potentially decrease wasting prevalence by a quarter (25%), translating to a reduction from 6.3% to 4.7%. This significant reduction in wasting is more pronounced in rural areas (29%) than in urban settings (7%). Conversely, a 14% increase in overweight prevalence was observed, with rural areas experiencing a higher rise (15%) than urban areas (13%). The study also highlighted variations in access to safe sanitation, improved water sources, healthcare services, income, maternal employment, and education levels, underscoring the complex interplay between these factors and malnutrition outcomes. Notably, the reduction in wasting prevalence was mainly attributable to input determinants rather than direct health inputs, suggesting the importance of broader socioeconomic factors in combating malnutrition. Achieving SDG targets presents a significant opportunity to mitigate wasting, particularly in rural communities. However, the uneven distribution of improvements underscores the need for targeted interventions in less affected areas. The concurrent rise in overweight prevalence, points to the emerging challenge of addressing the dual burden of malnutrition. This necessitates integrated, multi-sectoral strategies considering the diverse health determinants and nutritional status.

## Introduction

The United Nations member states came together in 2015 to endorse the 2030 Agenda for Sustainable Development, a collaborative roadmap toward achieving a better future for everyone on the planet [1]. The 17 Sustainable Development Goals (SDGs), along with their 169 associated targets, are a collective call for action dedicated to eradicating poverty and securing a peaceful and prosperous future for all by the year 2030 [1, 2].

Childhood malnutrition represents a significant global health challenge, predominantly affecting millions of young children under the age of five, especially in low- and middle-income countries [3]. In 2022, it was estimated that across the globe, 149 million children under the age of five were stunted, 45 million were wasted, and 37 million were overweight or obese [3]. It has a far-reaching impact on a child’s physical and cognitive development [4, 5]. Malnourished children have an increased risk of infectious and non-communicable diseases, and long-term sequelae include delayed mental development, poor school performance, and reduced intellectual capacity [6–8]. These impacts can extend into adulthood, affecting work capacity, causing reduced economic productivity and income, and intergenerational transmission of poverty and poor health [5, 6].

The unlikely coexistence of undernutrition and overweight, known as the double burden of malnutrition, which occurs at individual, household, or population levels and can manifest at different life stages, has been observed lately [9, 10]. At the individual level, various forms of malnutrition may occur during different life stages [9, 10]. In households, multiple members may concurrently experience different forms of malnutrition [9, 10]. Similarly, at the population level, various forms of malnutrition can be prevalent in specific geographic areas [9, 10].

The causes and factors leading to childhood malnutrition are complex and varied. They involve biological, environmental, social, economic, and political elements impacting individuals, households, and communities [5, 8, 11–14]. At the core of these determinants are maternal and child-specific factors. These factors include the mother’s level of education, income, employment status, diet, childbirth conditions, infant’s birth weight, duration and practice of breastfeeding, as well as water, sanitation, and hygiene facilities. [5, 8, 11–14]. In essence, the overall care that a child receives is essential for their nutritional well-being.

Building on UNICEF’s comprehensive malnutrition framework, which integrates health, education, and socio-economic factors, this study delves into the complex interplay between Sustainable Development Goals (SDGs) and childhood malnutrition [15]. Recognizing the multifaceted nature of the SDGs, which include both outcome indicators and variables influencing these outcomes, our research explores the tangible benefits of meeting specific SDG targets on child health. By modeling the effects of achieving these targets, the study illuminates the potential for significant improvements in child nutrition outcomes. Through this analysis, we aim to enrich the dialogue on nutrition and SDG synergies, offering valuable insights for policy formulation and implementation. Ultimately, the objective of the study was to evaluate the impact of achieving selected Sustainable Development Goal (SDG) targets on reducing childhood malnutrition, with a specific focus on wasting and overweight.

## Methods

### Study design and data sources

This research utilizes publicly available data from Demographic and Health Surveys (DHS) implemented by ICF International in 27 nations from 2015 to 2020. The DHS provides a snapshot every five years, capturing a broad spectrum of demographic, environmental, socioeconomic, nutritional, and health indicators, particularly from low- or middle-income countries. The methodology behind these surveys is standardized and uniform, ensuring comparability across different countries, with a notably high participant response rate. These surveys employ a stratified multistage cluster sample design, focusing on men and women aged 15 to 49 and children under five in selected households that constitute the primary sampling units [16]. The children’s recode (KR) dataset from the DHS of the 27 countries, which included information on 138,782 pairs of mothers and children, was analyzed for this study. Continuing our research series on malnutrition, the 27 countries included in this analysis had the full complement of required variables identified in our earlier publication [13]. This ensures consistency across our research and allows for a comprehensive examination of malnutrition.

### Outcome measures

We defined two binary outcome variables per the WHO’s Weight-for-Height growth standards, derived from Z-scores of weight and length measurements [17]. The first variable, ’wasted’, categorizes study participants as wasted if their Z-scores are below -2 standard deviations (SD) and not wasted otherwise. The second variable, ’overweight’, does similarly, classifying individuals as overweight if their Z-scores are above +2 SD. These thresholds align with the WHO guidelines, identifying children with Z-scores below -2 SD as moderately and severely wasted and those above +2 SD as overweight [18]. The weight-for-height measurement was favored for its robustness and reliability as an anthropometric measure [18]. We excluded children with missing or flagged weight-for-height Z-scores.

### SDG-related indicator variables

We included SDG-related indicators identified using the UNICEF conceptual framework on maternal and child nutrition, available as DHS dataset variables in our analysis [15]. These were categorized as health inputs: Access to health care services -SDG 3.8, improved water sources -SDG 6.1, and Safe Sanitation -SDG 6.2, which have a direct effect on the outcome variables. We also categorized health input determinants as the following: wealth index–SDG 1.1, maternal employment -SDG 8.5, and maternal education -SDG 4.1, which have an indirect effect on the outcomes variable and are mediated by the health inputs above. We used the wealth index constructed by the DHS; a proxy measure derived from asset ownership [19]. We also included innate predetermined child characteristics such as age, sex, and childbirth type in our analysis.

### Statistical analysis

We conducted a country-level descriptive analysis of key survey characteristics expressed as absolute numbers and percentages. In our analysis, variable distributions were calculated as means, taking into account the overall sample size. We conducted logistic regression for each simulation and applied the post-estimation command “mtable” to estimate marginal effects and average adjusted predictions. This facilitated the construction of predictive outcome tables based on both current and target values of the SDG-related indicators variables [20]. In accounting for the DHS survey design, we made appropriate adjustments to the analysis by factoring in the sample weights, stratification, and clustering.

Our simulations were based on the analytical framework of Glewwe and Miguel, 2007 and Beltrn and Castro, 2018 [21, 22]. The framework posits three distinct empirical specifications, each outlining unique health improvement scenarios.

The first scenario models child malnutrition as dependent on variables directly influencing nutrition and innate health attributes. This model that describes the relationship between child malnutrition and these direct factors is defined as a health production function. Within this framework, specific determinants, such as water, sanitation and hygiene, and healthcare access, modifiable by family choices, are referred to as health inputs. Other determinants, like the child’s age, childbirth type, and sex, are considered fixed. However, not all health inputs are quantifiable with DHS data; others are unobservable. These unobserved variables pose estimation challenges due to their likely correlation with observable inputs, as family decisions jointly determine both.

The second scenario is encapsulated in the demand function, illustrating the connection between child malnutrition and the determinants of health inputs. In response to the challenge posed by the unobservable variables, the demand function describes the relationship between child malnutrition and health input determinants: how much of the health inputs and child nutrition needs the family will be able to provide, given their socio-economic profile and other determinants of health, in addition to fixed factors that the family cannot change like the child’s age, childbirth type, and sex.

The third scenario, a hybrid production function, integrates both health inputs and health input determinants. It posits that input determinants should not factor into a production function if all health inputs are considered, though this is often impractical due to data constraints. A hybrid function compensates for unobserved inputs by substituting them with corresponding demand functions.

These functions underpinned our simulations and understanding of expected child malnutrition improvements when inputs and input determinants achieve specified targets. In estimating the production function, we regressed each outcome variable against innate predetermined child characteristics and health input variables. For the demand function, our outcome variables were regressed solely on the innate predetermined child characteristics and health input determinant variables separately. For the hybrid production function, the outcome variables were regressed on observed inputs, input determinants, and innate predetermined child characteristics. We set the observed inputs and input determinants from each of our function estimates at their target levels, taking the current mean value as a baseline to obtain our child malnutrition simulation predictions. We simulated predicted values for wasting and overweight separately and by place of residence and country.

Table 1 presents the aspirational target values for input and input determinant variables and the corresponding SDG target statements from which the target values were derived. These targets, uniformly set at 1, represent the achievement of the SDG goals’ targets.

**Table 1:** Target values for inputs and input determinants and their corresponding Sustainable Development Goals (SDGs).

Variable category	Variable	Proposed Target	Related SDG and targets
**Input**	Safe Sanitation	1.00	**SDG 6.2:** By 2030, achieve access to adequate and equitable sanitation and hygiene for all and end open defecation, paying special attention to the needs of women and girls and those in vulnerable situations
	Improved Water Sources	1.00	**SDG 6.1:** By 2030, achieve universal and equitable access to safe and affordable drinking water for all
	Access to Health Care Services	1.00	**SDG 3 Target 3.8:** Achieve universal health coverage, including financial risk protection, access to quality essential health-care services and access to safe, effective, quality and affordable essential medicines and vaccines for all.
**Input Determinants**	Maternal Employment	1.00	**SDG 8 Target 8.5:** By 2030, achieve full and productive employment and decent work for all women and men, including for young people and persons with disabilities, and equal pay for work of equal value
	Household Wealth Index	1.00	**SDG 1 Target 1.1**: By 2030, eradicate extreme poverty for all people everywhere, currently measured as people living on less than $1.25 a day
	Maternal Education (children whose mother’s highest educational attainment was secondary education or higher)	1.00	**SDG 4 Target 4.1:** By 2030, ensure that all girls and boys complete free, equitable and quality primary and secondary education leading to relevant and effective learning outcomes

All the analyses were performed using STATA16, and all charts and plots were drawn with R, a language and environment for statistical computing [23, 24].

### Ethics statement

This study is based on secondary datasets from the DHS; therefore, ethical approval was not required. We obtained approval from The DHS Program to download and use the datasets.

## Results

The pooled dataset from 27 countries had a total of 138,782 children under the age of five in 13,788 neighborhoods (Table 2). Among them, 6.3% suffered from wasting and 4.3% were overweight, and about two-thirds (67%) resident in rural neighborhoods. The mean prevalence of wasting among children in rural areas (6.5%) was slightly higher than in urban (5.7%) areas. Conversely, urban areas recorded a marginally higher rate of overweight children (4.7%) than rural areas (4.0%) (Table 3).

**Table 2:** Description of demographic and health survey data by countries, childhood malnutrition prevalence, and residence, 2015 to 2020.

Country	Survey Year	Number of Children	Number of Neighbourhoods	Wasted (%)	Overweight (%)	Residence (rural %)
**Albania**	2018	2,462	631	1.4	16.9	45.0
**Angola**	2016	6,407	625	5.0	3.6	40.0
**Armenia**	2016	1,561	304	4.2	13.5	43.6
**Benin**	2018	12,033	555	5.1	2.0	61.2
**Burundi**	2017	6,052	554	5.1	1.4	91.1
**Cameroon**	2019	4,477	428	4.4	11.1	55.3
**Gambia**	2020	3,811	279	5.3	2.3	34.2
**Guinea**	2018	3,430	399	9.1	6.0	71.8
**Haiti**	2017	5,598	449	3.8	3.6	66.5
**Liberia**	2020	2,457	324	3.7	4.5	48.3
**Malawi**	2016	5,178	850	2.8	4.5	87.1
**Maldives**	2017	2,362	260	9.2	4.1	69.2
**Mali**	2018	8,588	345	8.9	2.0	79.5
**Nepal**	2016	2,369	375	9.8	1.3	47.0
**Nigeria**	2018	11,405	1,378	6.9	2.1	55.9
**Pakistan**	2018	4,151	554	7.0	2.5	67.3
**Papua New Guinea**	2018	3,290	674	9.2	9.0	88.3
**Rwanda**	2020	3,809	500	1.2	5.8	83.3
**Senegal**	2019	5,531	214	8.0	2.4	63.6
**Sierra Leone**	2019	4,144	564	5.6	4.9	67.6
**South Africa**	2016	1,082	466	2.5	13.7	43.4
**Tajikistan**	2017	5,867	366	5.5	3.3	79.3
**Tanzania (United Republic of)**	2016	8,962	607	4.8	3.8	74.2
**Timor-Leste**	2016	5,718	455	24.2	5.4	71.3
**Uganda**	2016	4,413	688	3.8	4.0	79.7
**Zambia**	2019	8,711	545	4.3	5.3	65.1
**Zimbabwe**	2015	4,914	399	3.5	5.9	70.4
		**138,782**	**13,788**	**6.3**	**4.3**	**67.0**

**Table 3:** Summary of pooled sample characteristics of the demographic and health survey data.

Variables	Description	Mean		
		Overall	Residence	
			Urban	Rural
Outcomes: Child Malnutrition	1 Wasted 0 Otherwise	0.063	0.057	0.065
	1 Overweight 0 Otherwise	0.043	0.047	0.040
Predetermined child characteristics	Child’s Age (months)	28.4	28.4	28.4
	Child’s Sex (1 = Female)	0.494	0.492	0.495
	Childbirth Type (1 = part of multiple birth)	0.029	0.032	0.027
Inputs	1 Safe Sanitation 0 Otherwise	0.564	0.569	0.563
	1 Improved Water Sources 0 Otherwise	0.721	0.877	0.649
	1 Access to Health Care Services 0 Otherwise	0.620	0.776	0.543
Input Determinants	1 Maternal Employment (If Mother is employed) 0 Otherwise	0.587	0.571	0.594
	1 If Household Wealth Index (Index is equal or greater than the third quintile) 0 Otherwise	0.564	0.881	0.408
	1 Maternal Education (If Secondary or higher education is mother’s highest educational attainment) 0 Otherwise	0.351	0.546	0.255

The average age of the children in the pooled dataset was 28.4 months, and this was similar across urban and rural settings. The sex distribution was nearly even, with females representing 49.4% of the children overall. Interestingly, the proportion of children who were part of multiple births was slightly higher in urban areas (3.2%) than in rural ones (2.7%). Safe Sanitation was accessible to 56.4% of the population, with minimal variation between urban (56.9%) and rural (56.3%) areas. However, a significant disparity was observed in the access to improved water sources available to 87.7% of urban residents compared to only 64.9% in rural areas. Access to health care services showed a similar urban-rural divide, with 77.6% access in urban areas against 54.3% in rural areas. Maternal employment was 58.7%, with slightly more employed mothers in rural (59.4%) than in urban (57.1%) settings. A stark contrast was noted in household wealth, with 88.1% of urban households at or above the third wealth quintile, compared to 40.8% in rural households. A higher percentage of urban mothers had achieved secondary or higher education (54.6%) than their rural counterparts (25.5%).

The mean prevalence of wasting ranged from as low as 1.3% in Albania to a high of 24.1% in Timor-Leste (Table 4). In contrast, the occurrence of overweight children was most significant in Albania at 16.9%, with the lowest in Nepal at 1.2%. Demographically, the average child’s age across the countries studied had minimal variation; likewise, sex distribution was slightly skewed towards females. Instances of multiple births are relatively rare, hovering around 2% to 3%. Access to safe sanitation and improved water sources varied significantly by country, with Armenia reporting nearly universal access to improved water sources (98%), but only 41.3% to safe sanitation. Access to health care services ranged from over 85% in Albania to as low as 39.3% in Papua New Guinea. Maternal employment also varied, with high rates in countries like Benin (80.9%) and low in Pakistan (13.3%). The pooled dataset also showed disparities in maternal education levels, with the highest secondary or higher education attainment in Armenia (94.3%).

**Table 4:** Summary of country sample characteristics of the demographic and health survey data.

Country	Mean										
	Wasted	Overweight	Child’s Age (months)	Child’s Sex (Female)	Childbirth Type (part of multiple birth)	Safe Sanitation	Improved Water Sources	Access to Health Care Services	Maternal Employment	Household Wealth Index	Maternal Education–Secondary Education
Albania	0.013	0.169	29.033	0.495	0.025	0.139	0.830	0.852	0.309	0.555	0.502
Angola	0.050	0.036	27.730	0.500	0.024	0.327	0.523	0.438	0.742	0.546	0.311
Armenia	0.042	0.135	29.048	0.471	0.019	0.413	0.980	0.893	0.209	0.596	0.943
Benin	0.050	0.020	28.058	0.492	0.044	0.339	0.670	0.657	0.809	0.584	0.162
Burundi	0.051	0.014	28.948	0.498	0.021	0.764	0.810	0.676	0.882	0.575	0.108
Cameroon	0.044	0.111	28.213	0.486	0.043	0.661	0.709	0.578	0.689	0.540	0.401
Gambia	0.053	0.023	27.151	0.470	0.041	0.569	0.934	0.727	0.546	0.576	0.369
Guinea	0.091	0.060	28.156	0.489	0.031	0.551	0.774	0.505	0.650	0.529	0.124
Haiti	0.038	0.035	28.702	0.499	0.031	0.667	0.601	0.554	0.502	0.534	0.414
Liberia	0.037	0.045	27.412	0.501	0.022	0.253	0.797	0.686	0.648	0.519	0.374
Malawi	0.028	0.045	29.068	0.515	0.030	0.857	0.857	0.427	0.663	0.525	0.208
Maldives	0.092	0.041	30.817	0.492	0.015	0.092	0.998	0.697	0.357	0.553	0.807
Mali	0.089	0.020	27.866	0.493	0.031	0.636	0.671	0.696	0.577	0.580	0.149
Nepal	0.098	0.012	29.466	0.478	0.010	0.462	0.951	0.415	0.502	0.578	0.455
Nigeria	0.069	0.020	28.293	0.489	0.032	0.541	0.672	0.727	0.697	0.620	0.451
Pakistan	0.070	0.025	28.748	0.488	0.022	0.329	0.942	0.522	0.133	0.580	0.363
Papua New Guinea	0.092	0.090	29.664	0.464	0.022	0.449	0.430	0.393	0.308	0.596	0.255
Rwanda	0.012	0.058	28.755	0.500	0.026	0.835	0.789	0.759	0.745	0.566	0.231
Senegal	0.080	0.024	27.593	0.507	0.029	0.624	0.778	0.685	0.445	0.532	0.166
Sierra Leone	0.056	0.049	27.129	0.494	0.039	0.637	0.620	0.500	0.790	0.519	0.300
South Africa	0.025	0.137	29.213	0.493	0.032	0.194	0.895	0.758	0.273	0.526	0.886
Tajikistan	0.055	0.033	28.978	0.497	0.018	0.547	0.760	0.772	0.179	0.608	0.917
Tanzania (United Republic of)	0.048	0.038	27.528	0.493	0.030	0.697	0.551	0.530	0.787	0.536	0.140
Timor-Leste	0.241	0.054	29.948	0.499	0.015	0.276	0.804	0.522	0.334	0.601	0.569
Uganda	0.038	0.040	28.391	0.495	0.031	0.799	0.774	0.583	0.788	0.568	0.272
Zambia	0.043	0.053	28.315	0.503	0.026	0.780	0.672	0.661	0.470	0.523	0.385
Zimbabwe	0.035	0.059	28.913	0.508	0.030	0.748	0.740	0.606	0.404	0.556	0.666

### Simulation results for wasting

**Fig 1:**
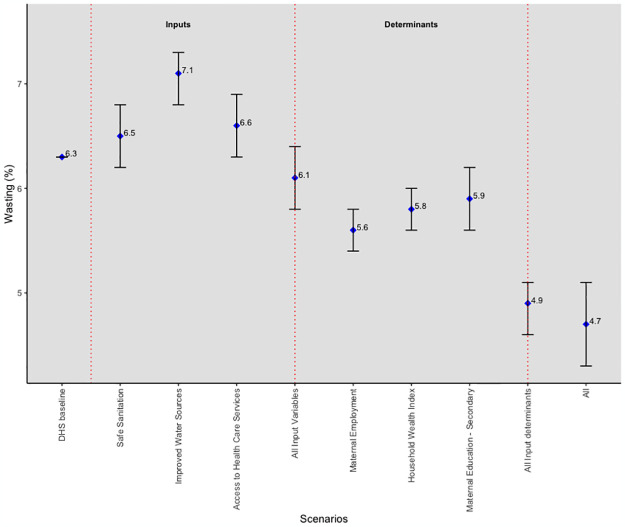
Simulation results for wasting.

The prevalence of wasting across the 27 countries was reduced by a quarter (25%), from 6.3% to 4.7%, a 1.6 percentage point difference, when all the SDG-related inputs and input determinants reached their target values (Fig 1). We obtained a similar reduction in the prevalence of wasting (4.9%) when all input determinants reached their targets. However, there was barely any decrease in the prevalence of wasting (6.1%) when all inputs reached their target values. A further examination of the predicted wasting prevalence shows that each input prediction at their respective target values was higher than the baseline. Therefore, it can be estimated that only 12.5% of the reduction in the prevalence of wasting was achieved through the effect of the included input variables, with the remaining 87.5% attributable to unincluded inputs produced when the input determinants reach their targets.

**Fig 2:**
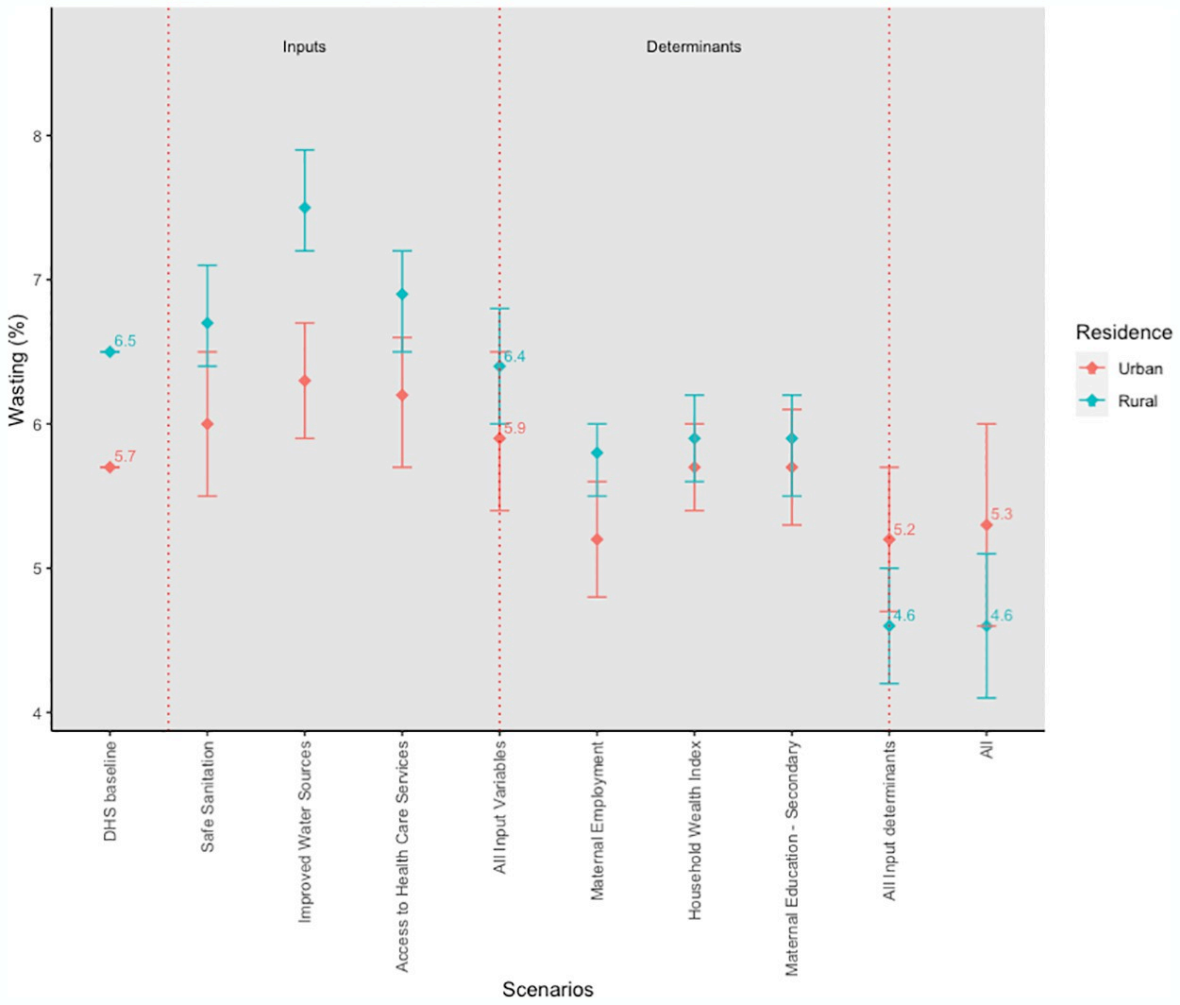
Simulation results for wasting by place of residence.

There was a predicted 7% reduction in the prevalence of wasting among residents of urban areas compared to the predicted 29% reduction in the wasting prevalence among residents of rural areas when all inputs and input determinants reached their target values (Fig 2). In rural areas, combined inputs and input determinants target values predictions were lower than the baseline, with the combined input determinants prediction lower than combined inputs. In urban areas, only the predicted combined prevalence at input determinants’ target values was lower than the baseline, with the predicted wasting prevalence at inputs’ target values just 0.2 percentage points higher than the baseline.

**Fig 3:**
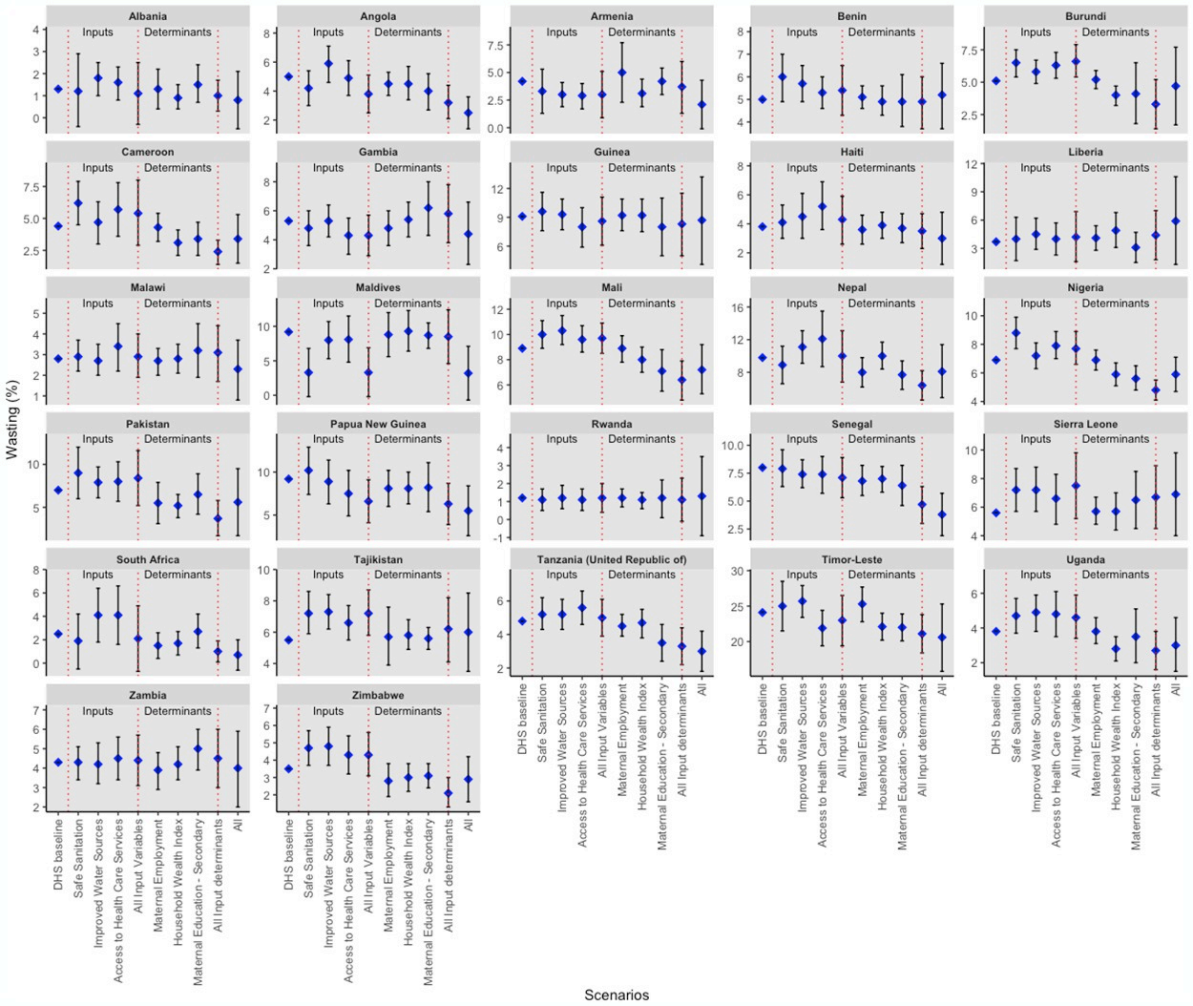
Simulation results for wasting by country.

Fig 3 provides a country-by-country breakdown of the wasting prevalence predictions when all the inputs and input determinants reached their target values. All countries except Benin, Liberia, Rwanda, Sierra Leone, and Tajikistan had a predicted reduction in the prevalence of wasting at target values. Notably, South Africa, Maldives, Senegal, Armenia, and Angola all had a greater than 50% reduction in child wasting prevalence, with 72.0%, 65.2%, 52.5%, 50%, and 50%, respectively. Guinea, Zambia, and Burundi had the slightest reduction in child wasting prevalence, with 4.4%, 7.0%, and 7.8%, respectively. Timor-Leste, despite having the highest wasting prevalence of 24.1, only had a predicted 14.5% reduction in child wasting prevalence. In stark contrast, Liberia and Sierra Leone saw a significant increase in predicted wasting prevalence, suggesting a worsening of wasting in the two countries. Furthermore, Benin, Rwanda, and Tajikistan also showed some increase in predicted child-wasting prevalence.

### Simulation results for overweight

**Fig 4:**
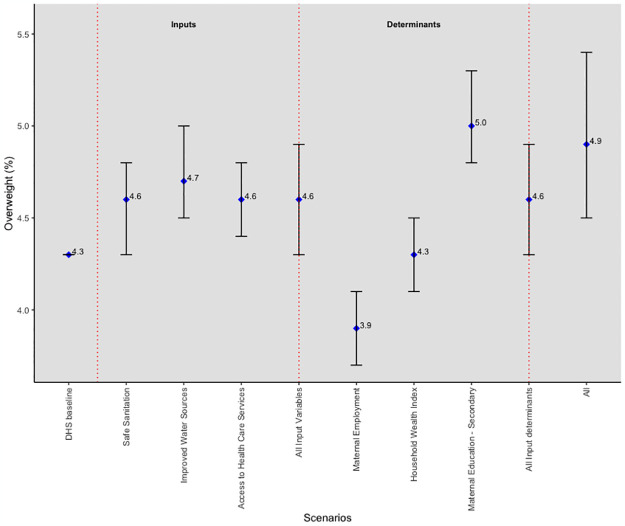
Simulation results for overweight.

At the target values of all the SDG-related inputs and input determinants, the prevalence of overweight increased from 4.3% to 4.9%, a 0.6 percentage point difference and a 14% change (Fig 4). A smaller percentage point difference (0.3) from the baseline was obtained when all inputs (4.6%) and input determinants (4.6%) reached their respective targets. A further examination of the predicted overweight prevalence shows that each input prediction at their respective target values had the same prevalence as the combined inputs prediction (4.6%) except for improved water sources, which was slightly higher at 4.7%. However, each input determinant prediction was lower than the combined input determinants’ prediction except for maternal education, which had the highest (5.0%) of all predictions across the board. An estimated 50% of the predicted increase in the prevalence of overweight was achieved through the effect of the included input variables, with the remaining 50% attributable to unincluded inputs produced when the input determinants reach their targets.

**Fig 5:**
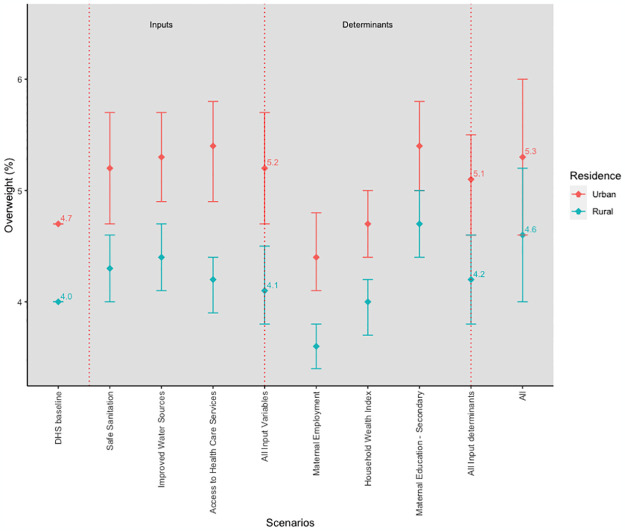
Simulation results for overweight by place of residence.

There was a predicted 13% increase in the prevalence of overweight among residents of urban areas compared to the predicted 15% increase among residents of rural areas when all inputs and input determinants reached their target values (Fig 5). In rural areas, combined inputs and input determinants target values predictions were 4.1% and 4.2% respectively. Similarly, in urban areas, both the combined inputs’ and input determinants’ target values predictions were higher than the baseline, with the combined inputs’ prediction slightly higher than that of the combined input determinants.

**Fig 6:**
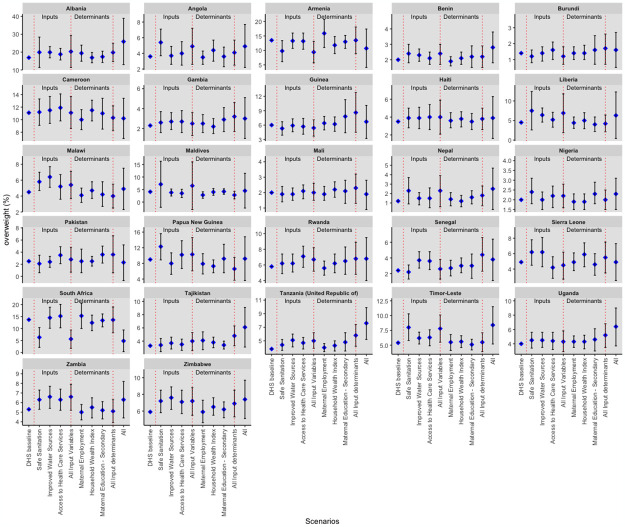
Simulation results for overweight by country.

Fig 6 provides a country-by-country breakdown of the overweight prevalence predictions when all the inputs and input determinants reached their target values. All countries except Armenia, Cameroon, Mali, Pakistan, Sierra Leone, and South Africa had a predicted increase in the prevalence of overweight at target values. Notably, Nepal, Tanzania, Tajikistan, Uganda, Senegal, Timor-Leste, and Albania all had a greater than 50% increment, with 108.3%, 100%, 84.8%, 60.0%, 58.3%, 55.6% and 53.3%, respectively. Papua New Guinea, Malawi, and Maldives had the least increment in prevalence, with 2.2%, 8.9%, and 9.8%, respectively. There was no change from baseline overweight prevalence in Sierra Leone. Conversely, we saw a significant reduction in overweight prevalence in South Africa and Armenia. Furthermore, Cameroon, Pakistan, and Mali showed some decrease in predicted overweight prevalence.

## Discussion

This analysis of the multi-country dataset encompassing 138,782 children under five years old across 27 countries and 13,788 neighborhoods found a 6.3% prevalence of wasting and a 4.3% incidence of overweight among the children. A higher rate of wasting was observed in rural neighborhoods (6.5%) compared to urban ones (5.7%), whereas urban neighborhoods had a slightly higher prevalence of overweight children (4.7%) than rural areas (4.0%).

Progress towards SDGs showed the potential to reduce the prevalence of wasting by a quarter (25%). This improvement was more pronounced in rural areas, which saw a predicted 29% reduction compared to a 7% reduction in urban areas. Not all countries benefited equally; South Africa, Maldives, Senegal, Armenia, and Angola could see reductions in child wasting by over 50%, while others like Guinea, Zambia, and Burundi might experience minimal reductions. Notably, some countries like Liberia, Sierra Leone, Tajikistan, Rwanda, and Benin could face an increase in wasting prevalence, highlighting the complex interplay of factors affecting wasting and the varying impacts of meeting SDG targets across different settings [25].

Previous studies on child nutrition trends and SDG attainment projections have buttressed our findings on the potential overall reduction in wasting prevalence when these selected SDG-related indicators improvement targets are met [21, 26, 27]. Childhood wasting is associated with low maternal education, poor household wealth, maternal unemployment, poor sanitation, unimproved water sources, and limited access to healthcare [12, 28–33]. This expected reduction was, however, only seen across some countries, with predicted significant increases in the prevalence of wasting in 5 countries. While this predicted significant increase in the prevalence of wasting in these five countries suggests the need for further investigation, examining these SDG-related indicators may attempt to explain these unexpected findings.

Two randomized controlled trials in Bangladesh and Kenya have disputed the relationship between improved drinking water sources and sanitation on child linear growth [34, 35]. This finding has been echoed by Johri et al. 2019 who also called into question the relationship between improved water sources and wasting and stunting [36]. Furthermore, the influence of maternal education on children’s nutrition appears to hinge on reaching a certain level of educational attainment [37–39]. Investigators in a case-control study have also found that maternal employment does not affect a child’s nutritional status [40]. Likewise, a community-based cross-sectional study found no significant difference in the nutritional status of children born to unemployed mothers and those in employment [41]. Conversely, maternal employment has been associated with increasing levels of childhood wasting and poor nutritional status, possibly due to reduced maternal contact and care time [42, 43].

While socioeconomic status remains a crucial determinant, about 60% of the association has been noted to be mediated through water, sanitation, and hygiene, which will require individual improvements and significant improvement in childhood wasting is only seen in per capita income and not by asset ownership [30, 44]. A previous study has suggested that earnings from mothers might be allocated predominantly for family assets rather than adequately providing for children’s nutritional needs [43]. Büttner et al. (2023) and Cermeño et al. (2023) provide further nuance, with the former finding a tenuous link between economic growth and wasting and the latter documenting the limited impact of sub-optimal healthcare systems in addressing child wasting [45, 46].

This study’s findings suggest a significant potential impact of achieving SDGs on the prevalence of overweight. We observed an increase in the prevalence of overweight children from 4.3% to 4.9%, marking a 14% rise. Geographically, the increase in overweight prevalence was more pronounced in rural areas (15%) compared to urban areas (13%). On a country level, while most countries saw an increase in overweight prevalence when targets were met, notable exceptions included Armenia, Cameroon, Mali, Pakistan, Sierra Leone, and South Africa, which predicted a decrease or no change. Specifically, Nepal, Tanzania, Tajikistan, Uganda, Senegal, Timor-Leste, and Albania could expect more than a 50% increase in overweight prevalence, underscoring the varied outcomes of achieving SDG targets on the prevalence of overweight across different regions.

The potential marginal increase in the prevalence of overweight with the achievement of these selected SDG-related indicators improvement targets from our findings has been supported by previous studies [47–49]. At the same time, the between-country differences, especially in the five countries where we found a reduction, may be accounted for by in-country socioeconomic characteristics and contexts that have been previously identified [48–52]. The effect of the socioeconomic profile, including education, employment, and living conditions, on childhood overweight has been shown to differ in magnitude between socioeconomic profile backgrounds across different countries [49, 50, 53, 54].

Paradoxically, children from lower socioeconomic profile backgrounds are at the most significant risk in high-income countries, while those from the highest socioeconomic profile backgrounds are at the most significant risk in low to middle-income countries [49, 51, 55]. This has been attributed to factors like limited access to healthy food and safe exercise options, lower emphasis on weight management, cultural norms about physical appearance, and barriers to socio-economic improvement in high-income countries, unlike low to middle-income countries where there is usually a considerable income inequality and consistent access to food is a challenge for those from lower socioeconomic profile backgrounds, and being overweight is often seen as an indicator of wealth [55]. Notably, the two countries (Armenia and South Africa) with the most significant reductions in the prevalence of childhood overweight were in the upper-middle-income group of countries. Cameroon, Mali, and Pakistan, with some reduction in prevalence, were in the low-income (Cameroon & Mali) and lower-middle-income (Pakistan) groups, respectively [56].

Recent research has highlighted the interactions within the SDGs, with most findings suggesting more positive synergies than negative trade-offs, and this provides further context for our findings [25, 57–59]. Multiple studies have noted the interdependencies and the dual role of SDGs as multipliers and buffers of the positive and negative correlations -highlighting SDGs 1 and 3 as key to the achievement of all others [59, 60]. SDGs 4 and 6 were also identified as multipliers of positive systemic effects while noting the potential of SDG 8 as a trade-off multiplier [60]. Notably, simulation has yet to demonstrate the complete attainment of all the SDGs [25]. These connections between SDGs have been highlighted to be highly dependent on various factors, including geographical location, governance structures, and socio-economic conditions, and these relationships vary significantly in different contexts [25]. These dynamics suggest the challenge in designing and implementing coherent policies and strategies for advancing SDGs and, by extension, improving child nutrition outcomes.

The preceding underscores the intricate challenges and varying successes in addressing wasting and overweight among children across diverse global regions. Our findings reflect the necessity for country-specific policy interventions and the importance of addressing underlying socioeconomic determinants that support healthy growth. We also provide valuable insights for policymakers by unpacking the relationship between childhood malnutrition and SDGs, illustrating the potential improvements through dedicated policy actions. A multifaceted approach, informed by robust data and cognizant of the diverse socioeconomic landscapes, is essential for improving global child nutrition.

Our study has some potential limitations worth noting. First, our cross-sectional study limits our ability to establish temporal relationships. Second, our selection of SDG indicators was limited by the availability of the related variables in the parent datasets. However, our simulations on the premise of target achievements of groups of SDG-related indicator variables tend to be less affected by biases often present in efforts to isolate the effect of a specific SDG-related indicator variable using observational data like the DHS [21]. Furthermore, this study consolidates data from Demographic and Health Surveys (DHS) that are nationally representative and widely applicable, covering 27 countries across five continents. Characterized by their high-quality data and high response rates, these surveys employ a rigorous methodology and well-documented data sources. The standardization of the DHS survey modules and their implementation enables the data to be comparable across the varied national landscapes.

## Conclusion

According to the multi-country study findings, there was a 6.3% prevalence of wasting and a 4.3% prevalence of overweight among children under the age of five, with rural neighborhoods showing a slightly higher rate of wasting and urban areas recording a marginally increased prevalence of overweight. The pursuit of SDGs offers substantial promise in reducing wasting (25%), particularly in rural settings. However, the distribution of these benefits is not uniform, suggesting that targeted interventions may be required in countries less impacted by SDG progress. The increase in overweight prevalence (14%), notably in rural areas, adds a layer of complexity to these challenges and highlights the need for integrated, multi-sectoral approaches to address the dual burden of malnutrition.

## Supporting information

S1 Dataset (DTA)
